# The NF-κB Nucleolar Stress Response Pathway

**DOI:** 10.3390/biomedicines9091082

**Published:** 2021-08-25

**Authors:** Hazel C. Thoms, Lesley A. Stark

**Affiliations:** The Stark Lab, Edinburgh Cancer Research Centre, Institute of Genetics and Cancer, University of Edinburgh, Crewe Rd. South, Edinburgh EH4 2XU, UK; H.Thoms@ed.ac.uk

**Keywords:** nucleolus, nucleoli, NF-κB, p62, nucleophosmin, NPM, COMMD1, RRN3, TIF-IA, aspirin, colon cancer

## Abstract

The nuclear organelle, the nucleolus, plays a critical role in stress response and the regulation of cellular homeostasis. P53 as a downstream effector of nucleolar stress is well defined. However, new data suggests that NF-κB also acts downstream of nucleolar stress to regulate cell growth and death. In this review, we will provide insight into the NF-κB nucleolar stress response pathway. We will discuss apoptosis mediated by nucleolar sequestration of RelA and new data demonstrating a role for p62 (sequestosome (*SQSTM1)*) in this process. We will also discuss activation of NF-κB signalling by degradation of the RNA polymerase I (PolI) complex component, transcription initiation factor-IA (TIF-IA (*RRN3*)), and contexts where TIF-IA-NF-κB signalling may be important. Finally, we will discuss how this pathway is targeted by aspirin to mediate apoptosis of colon cancer cells.

## 1. Introduction

Hyper-activation of the NF-κB pathway drives cancer progression and poses a serious threat to human health [1,2,3]. One mechanism that has emerged for controlling this activity is sequestration of NF-κB proteins in the nuclear compartment, the nucleolus [4,5]. Recent discoveries have extended our understanding of the mechanisms that control this nucleolar sequestration and the downstream consequences on cell phenotype. These studies highlight a role for the autophagic receptor p62 (sequestosome (*SQSTM1*)) in transporting NF-κB/RelA to nucleoli and, in particular, to intra-nucleolar aggresomes [6]. Remodelling of the nucleolus occurs as an early response to cell stress [7,8,9]. New data suggest that not only do nucleoli play a role in regulating the downstream transcriptional output of NF-κB, but that a specific, non-canonical form of nucleolar stress lies upstream of NF-κB, stimulating cytoplasmic to nuclear translocation of the transcription factor [10]. In this review, we will describe the various points of crosstalk between nucleoli and the NF-κB pathway and discuss the relevance of this NF-κB nucleolar stress response pathway in the regulation of cellular homeostasis, immunity, inflammation, and aging. We will also discuss the relevance to the treatment and prevention of cancer.

## 2. Regulation of Apoptosis by Nucleolar Sequestration of NF-κB/RelA

### 2.1. NF-κB and Disease

NF-κB is a family of highly conserved, inducible transcription factors that play a key role in regulating innate and adaptive immune responses, cellular homeostasis, and ageing [3,11,12,13]. The family comprise five members, namely, RelA (p65), RelB, c-Rel, p105/p50 (*NF-κB1*), and p100/p52 (*NF-κB2*). All members have a Rel homology domain which allows dimerization, translocation to the nucleus, and DNA binding, while only RelA, RelB, and C-Rel have the ability to drive transcription. The most abundant form of NF-κB is a heterodimer of the RelA/p50 subunits. In resting cells, this heterodimer is retained in the cytoplasm by the inhibitor protein, IκBα. If these cells are exposed to specific stimuli, including cytokines, bacterial pathogens, cytotoxic agents, nutrient deprivation, hypoxia, and physical insult, IκB is phosphorylated by inhibition of IκB (IKK) kinase complexes (IKK1/IKKα, IKK2/IKKβ IKKγ/Nemo). This phosphorylation targets IκB for ubiquitination and degradation by the 26S proteasome. NF-κB complexes are then free to translocate to the nucleus, where they influence the expression of a large and complex network of genes that regulate an array of cellular processes including proliferation, differentiation, inflammation and, apoptosis [14].

In healthy cells, the dynamics of NF-κB activation are tightly controlled by negative feedback mechanisms [15]. Hence, the duration of signalling is limited. However, in many human diseases including chronic inflammatory conditions, autoimmune disorders and age-related degenerative diseases, NF-κB signalling is constitutively active which is causally associated with disease progression [16,17]. NF-κB is also aberrantly active in the majority of human malignancies which stimulates cell growth, blocks apoptosis, perpetuates an inflammatory environment, accelerates cancer progression and conveys resistance to therapy [1,18]. Indeed, NF-κB is of paramount importance to mostly all the hallmarks of cancer and, as such, has been aggressively pursued as a therapeutic target [2,19,20].

While the main switch in NF-κB activation is degradation of IκB, the transcriptional output of NF-κB is also highly dependent on nuclear pathways of regulation [21,22]. These nuclear mechanisms of regulation are extremely important for cancer progression as they govern the genes that are activated or repressed in a specific context and hence, the downstream consequences on cell growth, death, differentiation, and inflammation. However, unlike cytoplasmic activation of NF-κB, nuclear pathways of regulation remain poorly understood. Mechanisms identified include post-translational modifications of NF-κB family members, and differential interaction with a plethora of coactivators, repressors, and chaperones, to form a variety of chromatin bound complexes [23,24,25]. One other mechanism that has emerged for regulating the nuclear activity of NF-κB, and inducing the apoptotic death of cancer cells, is nucleolar sequestration of NF-κB proteins in bodies known as nucleolar aggresomes [6,26].

### 2.2. Sequestration in Nucleoli Represses NF-κB Activity and Induces Apoptosis

The nucleolus is a highly dynamic, membrane-less nuclear organelle [7,27]. Although its most recognised role is the site of ribosome biogenesis, it is also a key player in stress response and the regulation of cellular homeostasis [28,29]. It is formed during late mitosis around tandem repeats of ribosomal DNA (rDNA) known as nucleolar organiser regions (NORs) [30]. Each nucleolus has a tri-partite organisation which is maintained through a combination of active rDNA transcription and liquid–liquid phase separation (LLPS): the fibrillar center (FC), where the RNA polymerase I machinery is active; the dense fibrillar component (DFC), that is enriched in fibrillarin; and the granular component (GC) that harbours B23 [8,9]. RNA polymerase I (PolI) activity is believed to occur at the interface between the FC and the DFC, while processing of newly synthesized ribosomal RNA (rRNA) and assembly with ribosomal proteins occurs within the GC [31]. When the cell is exposed to a wide array of stresses, this tri-partite structure is lost, rDNA transcription is inhibited, and there is a dynamic flux of proteins into and out of the organelle, which is ultimately responsible for the downstream effects on cell phenotype [7,32,33]. This process is broadly termed nucleolar stress and can take many forms, dependent on cell context (see below for more details).

Translocation into the nucleolus as a regulatory mechanism was first described in 1999, when it was discovered that CDC14 is temporarily sequestered in nucleoli to arrest cell cycle progression in yeast [34]. This phenomenon was termed nucleolar sequestration, which is now known to be an important mechanism for controlling gene expression and maintaining cellular homeostasis [35,36,37]. For example, dynamic nucleolar sequestration of RAG-1 has recently been shown to be a negative regulatory mechanism in V(D)J recombination [38]. MDM2 is sequestered in nucleoli in response to cellular stress, which prevents p53 degradation and consequently promotes cell cycle arrest and apoptosis (see below for details of this canonical nucleolar stress response pathway) [39,40]. The E3 ubiquitin ligase, von Hippel–Lindau protein (VHL), is another example of nucleolar sequestration [41]. VHL promotes the degradation of the transcription factor hypoxia-inducible factor (HIF) under normal oxygen conditions. Low extracellular pH triggers nucleolar sequestration of VHL, enabling HIF to evade degradation and promote transcription of target genes involved in oxygen homeostasis. In 2005, Stark et al. reported that nucleolar sequestration of RelA was also an important mechanism for regulating NF-κB activity and inducing apoptosis [26].

**Figure 1:**
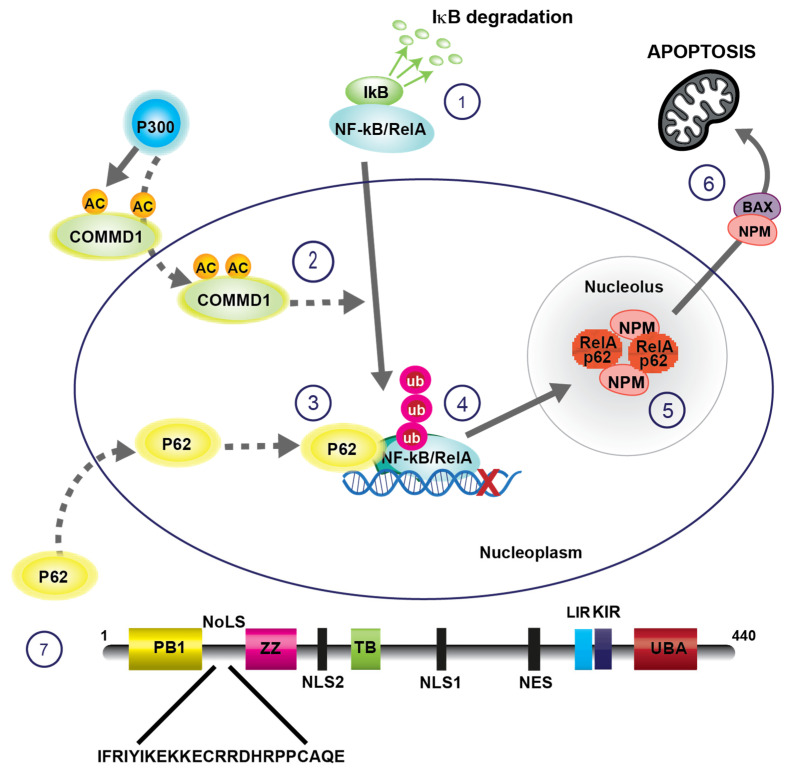
Nucleolar sequestration of RelA mediates apoptosis. 1. IκB degradation allows nuclear translocation of RelA/NF-κB complexes. 2. CommD1 is acetylated by p300, allowing it to bind RelA and promote its ubiquitination. 3 and 4. P62 accumulates in the nucleoplasm in response to specific stresses (including proteotoxic stress), binds RelA and other aggresome-related proteins and transports then to nucleolar aggresomes. 5 and 6. Nucleolar RelA causes translocation of nucleophosmin (NPM) to the cytoplasm. There it binds BAX and chaperones BAX to the mitochondria to mediate apoptosis. 7. Schematic of p62 protein domains showing the nucleolar localisation signal (NoLS). PB1-Phox and Bem1 domain; NLS-nuclear localisation signal; ZZ- Zinc finger domain; TB-TRAF6 binding domain; NES-nuclear export signal; LIR-LC3 interacting region; KIR-Keap interacting region; UBA-ubiquitin binding domain. Solid arrows depict main pathway. Dashed arrows represent recruited, facilitating proteins.

While exploring stress-mediated activation of the NF-κB pathway, it was noted that, in response to specific stress agents, RelA translocates from the cytoplasm to the nucleoplasm and then to nucleoli [26,42]. A nucleolar localisation signal (NoLS) was identified at the N-terminus of RelA and, using a dominant negative mutant deleted for this signal, it was shown that nucleolar sequestration of RelA is causally involved in repression of NF-κB-driven transcription and the induction of apoptosis (Figure 1). Since this initial report, nucleolar sequestration of RelA has been reported in response to chemopreventative agents (e.g., aspirin, sulindac, and sulindac suphone [26,43]), therapeutic agents (e.g., proteasome inhibitors, the anti-tumour agent 2-methoxyestradiol (2ME2), and TRK inhibition) [44,45]), stress inducers (e.g., UV-C radiation and serum starvation [26]), and expression of the homeobox transcription factor, Hox-A5 [46]. Interestingly, Campbell et al. (2021) recently explored the RelA interactome in response to DNA damaging agents and found that one of the top interacting clusters was proteins involved in ribosome biogenesis [24]. Nucleolar sequestration of p50 has also been demonstrated in response to the anti-TNF therapy, infliximab [47]. In contrast to stress inducers, RelA and p50 are retained in the nucleoplasm, excluded from nucleoli, in response to classical stimuli of the NF-κB pathway (such as TNF) that activate NF-κB-driven transcription and inhibit apoptosis [26]. Understanding the molecular signals that regulate the nuclear distribution of RelA would be highly advantageous as it may reveal a new category of therapeutics that eliminate diseased cells by targeting hyperactive nuclear RelA to the nucleolus.

### 2.3. COMMD1-Mediated Ubiquitination of RelA Signals for Nucleolar Translocation

The exact mechanisms that control nucleolar sequestration of regulatory proteins remains poorly understood. One mechanism that has been proposed is detention of proteins in “nucleolar detention centres” by stress-inducible, long noncoding RNA derived from the ribosomal intergenic spacer region [48]. Proteins detained by this mechanism encode a specific nucleolar detention sequence (NoDS) and include VHL, HSP70, MDM2/PML, and POLD1 [49]. Nucleolar detention centres were recently found to have amyloidogenic-like characteristics, therefore NoDS were renamed “amyloid converting motifs” (ACM) and detention centres renamed “amyloid bodies” or A-bodies [35,37]. Despite extensive analysis, RelA was not found to have an ACM (unpublished data), ruling out this mechanism of detention.

Another pathway that has been implicated in nucleolar sequestration is the ubiquitin-proteasome system. Upon chemical proteasome inhibition there is an influx of proteasome target proteins into nucleoli (including oncogenes, tumour suppressor genes, cyclin dependent kinases, and translation factors) that accumulate in bodies known as nucleolar aggresomes. These bodies bare similarity to cytoplasmic aggresomes observed in neurodegenerative disorders in that they are rich in heat shock factors and ubiquitinated and sumoylated proteins, and are linked to abrogated protein degradation [35,50,51,52]. They are generally seeded by RNA, although the exact roles and identities of the RNA species are still not clear. Interestingly, recent reports suggest nucleoli not only sequester ubiquitinated proteins, but may also act as a site of protein degradation [53].

In 2010 Thoms et al. demonstrated that RelA accumulates in nucleoli in response to proteasome inhibition suggesting ubiquitination may also play a role in nucleolar transport of this protein [54] (Figure 1). Indeed, the group went on to show that RelA is ubiquitinated in an NoLS-dependent manner in response to specific stresses and proteasome inhibition [54]. The multi-functional protein, COMMD1 (*MURR1*), had previously been shown to ubiquitinylate RelA in the nucleus in response to cytokine (e.g., TNF) stimulation [55,56,57,58,59]. This ubiquitination resulted in proteasomal degradation of RelA. Thoms et al. found that COMMD1 also ubiquitinates RelA in response to stress inducers. However, this ubiquitination resulted in nucleolar sequestration of RelA and apoptotic cell death [54]. In subsequent studies it was shown that COMMD1 is acetylated by p300 in response to stress, but not in response to TNF (Figure 1) [60]. It was also shown that p300-mediated acetylation of COMMD1 is essential for stress-induced ubiquitination and nucleolar translocation of RelA. These data suggest the intriguing possibility that COMMD1 acetylation, through differential ubiquitination, controls the nuclear distribution of RelA and the consequent downstream effects on cellular homeostasis.

### 2.4. RelA Is Sequestered in Nucleolar Aggresomes

Despite the dynamic nature of the nucleolar proteome, proteins sequestered in A-bodies and aggresomes are generally present in a solid, immobile state [35,49,61,62,63,64]. RelA accumulates in specific foci within the nucleolus in response to stress inducers and proteasome inhibition, suggesting it may also be present in immobile bodies. Lobb et al. recently tested this hypothesis using focal correlation spectroscopy (FCS) [6]. This technique is ideal for such studies as it can report molecular concentrations and diffusion rates of proteins at multiple points within a cell at a microsecond timescale [65]. These studies revealed that RelA motility increases in the cytoplasm in response to stress, in keeping with release from IκB, while the motility of the protein is significantly reduced in nucleoli, in keeping with retention in immobile bodies. Stable Isotope Labelling of Amino acids in Culture (SILAC)-based quantitative proteomics and immunocytochemistry revealed that RelA accumulates in nucleoli alongside heat shock proteins, translation factors and proteins of the ubiquitin-proteasome system, which, as outlined above, are typical components of nucleolar aggresomes [6]. It was concluded from this series of studies that RelA is ubiquitinated in response to specific stresses in a manner dependent on p300-acetylated COMMD1, and that this ubiquitination targets the protein to nucleolar aggresomes, causing a reduction in NF-κB-driven transcription and apoptotic cell death (Figure 1).

### 2.5. P62 Transports Ubiquitinated RelA to Nucleolar Aggresomes

How ubiquitinated proteins, including RelA, are transported from the nucleoplasm to nucleolar aggresomes remained unclear until a recent report uncovered a novel role for p62 [6]. P62 is a multifunctional protein that acts as a signalling scaffold and autophagy cargo receptor [66,67]. It colocalises with cytoplasmic aggregates and plays a significant role in their clearance [68]. It also shuttles to the nucleus, although its role in this compartment is still not clear [69]. It interacts with the autophagosomal marker, LC-3B I/II, which has previously been shown to be present in nucleoli [70]. It is known to interact with RelA and reportedly transports RelA to autophagosomes for degradation [71].

A recent study from our group demonstrated that p62 rapidly translocates to the nucleus and then to the nucleolus in response to the model stress inducer, aspirin, and the proteasome inhibitor, MG132 (Figure 1) [6]. The dynamics of this nucleolar translocation paralleled that of RelA. Furthermore, the two proteins co-localised in foci in the compartment. Inhibition of autophagy (using BafilomycinA) had minimal influence on the nuclear distribution of RelA or p62, ruling out the possibility that nucleolar sequestration is a consequence of altered autophagic activity. To translocate to autophagosomes, p62 firstly must homodimerize, which is dependent on the N-terminal PB1 domain (Figure 1) [69]. Surprisingly, the PB1 domain is dispensable for nucleolar localisation of p62, as is the ubiquitin binding domain (UBA) and the LC3 interacting region (LIR) [6]. Rather, a 22 amino acid nucleolar localisation signal was identified at the N-terminus of p62 that is absolutely essential for transport to nucleoli under conditions of cell stress (Figure 1) [6]. Using a dominant negative mutant deleted for this domain (p62∆NoLS), it was shown that inhibiting p62 nucleolar transport blocks nucleolar sequestration of RelA and the aggresome protein, SUMO-2/3. In contrast, blocking nucleolar translocation of RelA had no effect on stress-induced nucleolar sequestration of p62. Given that p62 transports ubiquitinated proteins to nucleolar aggresomes, it would be predicted that the UBA domain of p62 is essential. However, the role of the UBA domain in nucleolar transport of RelA has yet to be confirmed. Together, these data indicate a novel role for p62 in trafficking nuclear proteins to nucleolar aggresomes under conditions of stress (Figure 1). They also provided invaluable information on the mechanisms that regulate the nuclear/nucleolar distribution of RelA that could be exploited for therapeutic purpose.

### 2.6. RelA-Nucleophosmin Signalling

It was originally presumed that nucleolar RelA causes apoptosis of cancer cells as the protein is sequestered away from promoters of pro-proliferation and anti-apoptotic genes. However, use of a nucleolar targeting construct (RelA fused to the NoLS of the HIV-rev protein) revealed that specifically targeting RelA to this compartment triggers a cascade of events that actively induces apoptosis (Figure 1) [72]. Central to this pro-apoptotic pathway is the nucleolar phospho-protein nucleophosmin (NPM)/B23. Although NPM is predominantly nucleolar, it can also be found in both the nucleoplasm and cytoplasm, and is known to regulate multiple signalling networks [73]. In terms of NF-κB, NPM can act as a co-activator at specific gene sites and, in specific contexts, chaperones NF-κB to the nucleus to initiate transcription [74,75]. Reciprocally, RelA can also influence the activity of NPM. It was shown that nucleolar RelA causes NPM to relocate from the nucleolus to the cytoplasm, bind BAX, then transport BAX to the mitochondria to initiate apoptosis [72,76,77,78]. Others have also demonstrated NPM relocalisation and an NPM-BAX interaction in response to stress stimuli that induce nucleolar translocation of RelA (e.g., UV-C radiation) [77].

Whilst expression of the p62∆NoLS mutant inhibits nucleolar translocation of RelA and aspirin-mediated apoptosis, our studies indicate it has no effect on aspirin-mediated repression of NF-κB-driven transcription [6]. This is not surprising as ubiquitination renders RelA inactive [55,79] and it is likely that expression of the p62 NoLS deletion mutant causes accumulation of ubiquitinated RelA complexes at target promoters. What is surprising is that this repression is not sufficient to induce apoptosis, which would suggest the majority of the apoptotic effect of nucleolar translocation of RelA comes from activation of nucleolar, pro-apoptotic pathways.

## 3. Nucleolar Disruption Lies Upstream of NF-κB Pathway Activation in Response to Stress

**Figure 2:**
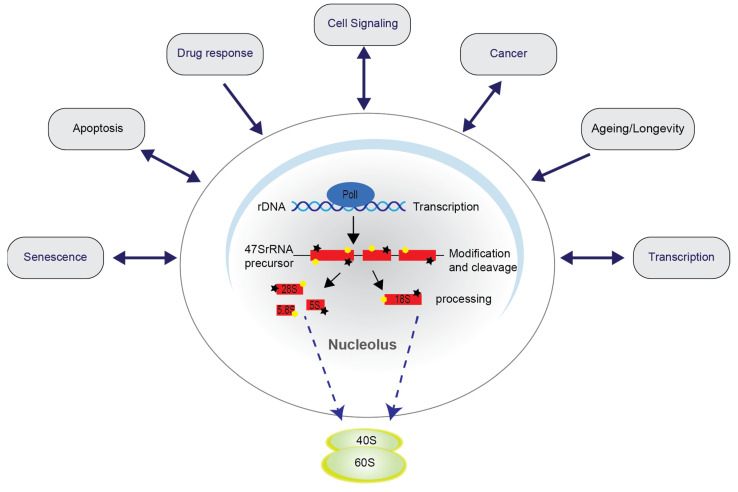
Schematic demonstrating the nucleolus as the hub of ribosome biogenesis and as a critical regulator of cellular homeostasis. Ribosome biogenesis starts with transcription of the 47S pre ribosomal RNA (rRNA) by the PolI preinitiation complex. The 47S transcript is cleaved, processed and packaged to eventually generate the 40S and 60S ribosomal sub-units. The function of the nucleolus is altered by a plethora of environmental and oncogenic stresses. This change in nucleolar function alters numerous cellular processes to alter phenotype and regulate homeostasis. Dashed lines represent movement within the cell. Black stars and yellow dots represent assembly factors. Arrows depict the direction of influence.

**Figure 3:**
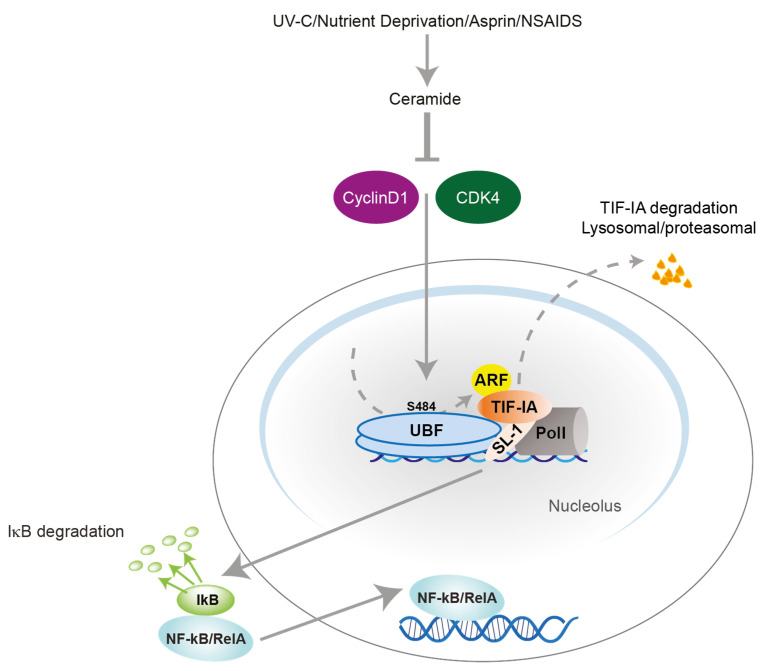
The TIF-IA-NF-κB nucleolar stress response pathway. When cells are exposed to specific stimuli, a cascade of events is triggered that leads to degradation of transcription initiation factor IA(TIF-IA) in a manner dependent on the second messenger ceramide, inhibition of cyclin dependent kinase 4 (CDK4) (depicted by T shaped line), recruitment of the nucleolar protein p14ARF (ARF) and the RNA polymerase I (PolI) complex component, upstream binding factor (UBF). Specifically, serine (s) 484 of this protein. This TIF-IA degradation results in degradation of IκBα, which allows NF-kB to translocate to the nucleus and regulate expression of target genes that control cellular processes such as apoptosis, proliferation, differentiation and senescence. Solid arrows depicts pathway to NF-κB nuclear translocation. Dashed arrows depict TIF-IA degradation pathway. NSAIDs: non-steroidal anti-inflammatory drugs.

Ribosome biogenesis is a well-orchestrated process that is the most energy consuming in the cell and, as such, is tightly linked to metabolic and proliferative activity [7,80]. The rate limiting step is transcription of ribosomal DNA (rDNA), which is mediated by the pre-initiation complex (PIC), consisting of the primary components: upstream binding factor (UBF), SL-I, transcription initiation factor-IA (TIF-IA (*RRN3*)), and PolI (Figure 2 and Figure 3) [81,82]. If cellular homeostasis is disrupted, for example by nutrient deprivation, exposure to cytotoxic agents, viral infection, or oncogene inactivation, PolI-driven transcription is rapidly downregulated, nucleolar stress pathways are activated, and a cascade of signalling events is triggered that influences cell phenotype [28,83,84,85]. Processes altered as a consequence of nucleolar stress include differentiation, metabolism, cell cycle progression, autophagy, senescence and, as outlined above, apoptosis (Figure 2 and Figure 3). Indeed, perturbations in nucleolar function are associated with many common diseases, including ischaemic heart disease, neurodegenerative disorders, and cancer.

The most recognised effector of nucleolar stress is the L5/11-MDM2-p53 pathway [86,87,88]. However, a number of p53-independent pathways have emerged that play an equally critical role in the maintenance of cellular homeostasis downstream of nucleolar disruption [87,89]. For example, Pecoraro et al. recently demonstrated that a G-Quadruplex TBA derivative causes nucleolar stress, cell cycle arrest, and apoptosis in a manner independent of p53 but dependent on the ribosomal protein UL3 [90]. In another study, the Russo lab demonstrated that mimicking perturbed ribosome biogenesis, in the absence of p53, caused the formation of an RpL3-Sp1-NPM complex at the p21 promoter and, consequently, cell cycle arrest and apoptosis [91]. Increasing evidence suggests that NF-κB signalling also lies downstream of nucleolar disruption, which may play an important role in innate immunity, senescence, and maintenance of cellular homeostasis.

### 3.1. TIF-IA-NF-κB Nucleolar Stress Response

The mechanisms by which multiple heterogeneous stresses converge on the NF-κB pathway have been far from clear but, given the parallels between stresses that disrupt ribosome biogenesis and those that activate NF-κB, nucleolar disruption could provide a unifying mechanism [4]. Our lab recently explored this possibility and uncovered a novel nucleolar stress response pathway. This pathway is dependent on the PIC component, TIF-IA, and lies upstream of IκB degradation. We have named this novel signalling axis the TIF-IA-NF-κB nucleolar stress response pathway (Figure 3) [10].

TIF-IA is an essential component of the PIC as it recruits PolI to the rDNA promoter [81,92,93]. In response to environmental perturbations, TIF-IA is targeted by a plethora of signalling pathways (e.g., mTOR, AMPK, ERK, and PP2A) which alters its phosphorylation status to fine-tune the PolI transcriptional output [93,94,95]. Although microscopically it appears to be mainly nucleolar, Szymanski et al. found that the majority of the protein is in the cytoplasm and that it shuttles rapidly between there, the nucleoplasm, and nucleoli [96]. Genetic deletion of TIF-IA in mice causes embryonic lethality [97]. Deletion or depletion in mouse embryonic fibroblasts (MEFs), cancer, and neuronal cells induces nucleolar segregation, suggesting the protein plays a role in maintaining the structure of the organelle (see below) [9,96]. Depletion also induces cell cycle arrest and apoptosis, indicating TIF-IA inactivation has important downstream consequences on cell phenotype [93,98,99].

Chen et al. found that multiple stress stimuli of NF-κB, including aspirin, UV-C, and the second messenger ceramide, not only alter the phosphorylation status of TIF-IA, but also induce degradation of the protein (Figure 3) [10]. This effect lies downstream of CDK4 inhibition, which is a common response to stress stimuli of the NF-κB pathway [100]. It is also specific, as it is not observed in response to TNF or the DNA damaging agent, camptothecin. It is dependent on both lysosomal and proteasomal degradation, but is distinct from the previously reported pathway to TIF-IA degradation that is facilitated by the E3 ligase, MDM2 [94]. It was shown that TIF-IA is dephosphorylated at Serine 44 in response to specific stress stimuli and that this dephosphorylation, along with the PolI complex associated factors UBF and p14ARF, are essential for TIF-IA degradation (Figure 3).

Characteristic consequences of nucleolar stress are inhibition of ribosomal DNA (rDNA) transcription, a reduction in nucleolar size and segregation of the FC and DFC to nucleolar caps at the periphery of the organelle [7,86]. Stress-mediated degradation of TIF-IA also causes inhibition of rDNA transcription and segregation of nucleolar marker proteins [10]. However, uncharacteristically, it causes a reduction in nucleolar number and a significant increase in nucleolar size. These changes are suggestive of altered nuclear biophysics and merging of the organelles. An increase in nucleolar volume, associated with altered rRNA transcription and nucleolar architecture, has also been observed in response to the proteasome inhibitor, MG132 [101], and the NEDD8 inhibitor, MLN4924 [102].

The first indication that nucleolar disruption stimulates NF-κB signalling came from studies demonstrating that siRNA depletion of TIF-IA (or other components of the PolI complex) activates the cytoplasmic NF-κB pathway, as indicated by degradation of IκB, phosphorylation of RelA at the critical S536 residue, nuclear translocation of NF-κB/RelA, increased NF-κB-driven transcription, and increased transcription of NF-κB target genes (Figure 3) [10]. This effect was independent of rDNA transcription as it was not mimicked by potent PolI inhibitors (CX5461, BMH21, and ActD). Stress effects on TIF-IA stability and nucleolar morphology preceded degradation of IκB and nuclear/nucleolar translocation of RelA. Furthermore, siRNA, genetic mutants and chemical inhibitors indicated that blocking TIF-IA degradation not only blocked stress effects on nucleolar size, but also blocked IκB degradation, nuclear/nucleolar translocation of RelA and apoptosis [10] (Figure 3). The link between TIF-IA degradation and NF-κB pathway stimulation was confirmed using explants of human colorectal tumours treated ex vivo with low dose aspirin. These studies revealed a very strong inverse correlation between tumour levels of TIF-IA following aspirin exposure (as indicated by Western blot analysis) and activation of the NF-κB pathway (as indicated by quantitative immunohistochemistry for RelA^p536^) [10]. Together, these results demonstrated for the first time that nucleolar disruption can stimulate pathways upstream of IκB degradation/NF-κB nuclear translocation to regulate cellular homeostasis.

Although the signalling networks that link TIF-IA to IκB degradation are not yet clear, a number of nucleolar shuttling proteins are known to impact NF-κB signalling. The most promising candidate is CK2, as it binds TIF-IA at the PolI complex [95] and facilitates IκB phosphorylation/degradation in response to UV-C radiation [103]. eIF2α is also known to modulate both PolI and NF-κB activity in response to ER stress [104,105,106]. Ribosomal proteins themselves can regulate NF-κB signalling. The ribosomal protein L3 reportedly binds to and stabilises IκB, thus repressing NF-κB activity [107,108]. In contrast, S3 promotes activity by interacting with NF-κB complexes in the nucleus [109]. An interesting new study by Beji et al. demonstrated that NPM is secreted from cardiac mesenchymal progenitor cells in response to doxorubicin and UV-C-mediated nucleolar stress [110]. They also demonstrated that the secreted NPM binds Toll-like receptor 4 (TLR4), thus inducing cytoplasmic to nuclear translocation of NF-κB and a pro-inflammatory phenotype. Further research into the links between nucleoli and NF-κB will help us fully understand how both pathways regulate cellular homeostasis under normal and stress conditions and reveal potential new targets for anti-inflammatory agents.

### 3.2. TIF-IA-NF-κB Nucleolar Stress Response in Senescence and Ageing

One other scenario where enlarged nucleoli are associated with NF-κB pathway activation is in cellular senescence, which is a state of permanent cell cycle arrest induced by DNA damage (e.g., telomere shortening that leads to replicative senescence) and other stresses (e.g., activated oncogenes- oncogene induced senescence (OIS)) [111,112]. It plays an important role in development, tissue remodeling, and cancer, and contributes to organismal aging [113,114]. A striking characteristic of senescence is a huge increase in nucleolar size and a reduction in number [115]. Another important characteristic is the NF-κB-dependent senescence associated secretory phenotype (SASP), which can reinforce cell cycle arrest, lead to paracrine senescence, promote the activation of senescence surveillance and also promote tumour progression [112]. Given the similarity between the phenotype of senescent cells and those showing loss of TIF-IA, it would be interesting to speculate that TIF-IA-NF-κB nucleolar stress plays a role in senescence. In keeping with a link between the two pathways, Nishimura overexpressed TIF-IA in IMR90 fibroblasts to mimic altered rDNA transcription and observed an increase in nucleolar size, activation of NF-κB, and an increase in SA-β-Gal positive cells (a marker of senescence) [116]. Nucleolar enlargement, such as that seen in response to altered TIF-IA levels, has recently been identified as a hallmark of aging tissue [113,114], as is chronic activation of the NF-κB pathway (inflammaging) [117].

## 4. Aspirin Acts against Colon Cancer Cells by Targeting the NF-κB Nucleolar Stress Response Pathway

Colorectal cancer remains the third most common cause of cancer death worldwide, with 1.93 million deaths annually (https://www.who.int/news-room/fact-sheets/detail/cancer) (accessed on 18 August 2021). Early detection and prevention are widely recognised to be key in combatting this disease. Aspirin and related non-steroidal anti-inflammatory drugs (NSAIDs) are one group of agents that have shown remarkable potential in the prevention of colorectal cancer [118,119,120]. Indeed, it has been suggested that long term aspirin use could lower colon and rectal cancer risk by up to 50%. However, the side effect profile of aspirin limits its use in the general population. Understanding the mechanisms by which aspirin acts against colon cancer cells is now paramount, as it would reveal biomarkers that could be used to improve the benefit to risk ratio, especially for patients diagnosed with pre-cancerous lesions.

In our lab, we have used aspirin extensively as a tool to explore nucleolar-NF-κB signalling [121]. These studies revealed that aspirin (at pharmacological doses) causes degradation of TIF-IA and inhibition of rDNA transcription, both in vitro and in explants of colorectal tumours treated ex vivo with the agent [10] (Figure 3). They also revealed that this TIF-IA degradation causes nuclear translocation of NF-κB/RelA, which is essential for the apoptotic effects of the agent [42]. COMMD1 levels are reduced in multiple cancer types, which contributes to invasion and progression [122]. In independent studies, we demonstrated that aspirin causes an increase in levels of COMMD1 through p300 acetylation [54,60] (Figure 1). We have also shown that this increased COMMD1 facilitates ubiquitination of newly translocated RelA/NF-κB which, alongside p62, targets RelA to nucleolar aggresomes [6,54] (Figure 1). The presence of RelA in the nucleolus kills colon cancer cells thorough a nucleophosmin-dependent pathway [72] (Figure 1).

Uncontrolled rDNA transcription is a hallmark of colorectal cancer and contributes to tumour growth by allowing de-regulated protein synthesis and uncontrolled activity of nucleolar cell growth/death pathways [123,124]. Uncontrolled NF-κB activity is also a hallmark of colorectal cancer, which drives disease progression by promoting inflammation and directing expression of pro-growth and anti-apoptotic genes [125]. We propose that aspirin inhibits both these driver mechanisms by targeting TIF-IA (causing reduced rDNA transcription and nuclear translocation of NF-κB/RelA) and COMMD1 (which ubiquitinates newly translocated RelA, facilitating RelA nucleolar translocation, reduced NF-κB-driven transcription and cell death). Indeed, our studies suggest that these two proteins may act as viable biomarkers for aspirin response and targets for novel chemopreventative agents.

## 5. Summary

Accumulating evidence suggests that there are several points of cross talk between nucleoli and the NF-κB pathway, and that this crosstalk may be important in regulating cellular homeostasis and the anti-tumour effects of aspirin. Further understanding of the NF-κB nucleolar stress response pathway is very important as it could reveal targets for agents that would be of use in multiple diseases associated with chronic NF-κB activity as well as biomarkers for cancer progression, aspirin response, and ageing.

## Data Availability

Not applicable.

## References

[1] Taniguchi K., Karin M. (2018). NF-κB, inflammation, immunity and cancer: Coming of age. Nat. Rev. Immunol..

[2] Prescott J.A., Cook S.J. (2018). Targeting IKKbeta in Cancer: Challenges and Opportunities for the Therapeutic Utilisation of IKKbeta Inhibitors. Cells.

[3] Perkins N.D. (2012). The diverse and complex roles of NF-κB subunits in cancer. Nat. Rev. Cancer.

[4] Chen J., Stark L.A. (2019). Insights into the Relationship between Nucleolar Stress and the NF-κB Pathway. Trends Genet..

[5] Chen J., Stark L.A. (2018). Crosstalk between NF-κB and Nucleoli in the Regulation of Cellular Homeostasis. Cells.

[6] Lobb I.T., Morin P., Martin K., Thoms H.C., Wills J.C., Lleshi X., Olsen K.C.F., Duncan R.R., Stark L.A. (2021). A Role for the Autophagic Receptor, SQSTM1/p62, in Trafficking NF-κB/RelA to Nucleolar Aggresomes. Mol. Cancer Res..

[7] Boulon S., Westman B.J., Hutten S., Boisvert F.M., Lamond A.I. (2010). The Nucleolus under Stress. Mol. Cell.

[8] Mangan H., Gailin M.O., McStay B. (2017). Integrating the genomic architecture of human nucleolar organizer regions with the biophysical properties of nucleoli. FEBS J..

[9] Nemeth A., Grummt I. (2018). Dynamic regulation of nucleolar architecture. Curr Opin Cell Biol..

[10] Chen J., Lobb I.T., Morin P., Novo S.M., Simpson J., Kennerknecht K., von Kriegsheim A., Batchelor E.E., Oakley F., Stark L.A. (2018). Identification of a novel TIF-IA-NF-κB nucleolar stress response pathway. Nucleic Acids Res..

[11] DiDonato J.A., Mercurio F., Karin M. (2012). NF-κB and the link between inflammation and cancer. Immunol. Rev..

[12] Gilmore T.D. (2006). Introduction to NF-κB: Players, pathways, perspectives. Oncogene.

[13] Cartwright T., Perkins N.D., C L.W. (2016). NFKB1: A suppressor of inflammation, ageing and cancer. FEBS J..

[14] Pahl H.L. (1999). Activators and target genes of Rel/NF-κB transcription factors. Oncogene.

[15] Ankers J.M., Awais R., Jones N.A., Boyd J., Ryan S., Adamson A.D., Harper C.V., Bridge L., Spiller D.G., Jackson D.A. (2016). Dynamic NF-κB and E2F interactions control the priority and timing of inflammatory signalling and cell proliferation. Elife.

[16] Tak P.P., Firestein G.S. (2001). NF-κB: A key role in inflammatory diseases. J. Clin. Investig..

[17] Mankan A.K., Lawless M.W., Gray S.G., Kelleher D., McManus R. (2009). NF-κB regulation: The nuclear response. J. Cell Mol. Med..

[18] Dolcet X., Llobet D., Pallares J., Matias-Guiu X. (2005). NF-κB in development and progression of human cancer. Virchows Arch..

[19] Kim H.J., Hawke N., Baldwin A.S. (2006). NF-κB and IKK as therapeutic targets in cancer. Cell Death Differ..

[20] Hayden M.S., Ghosh S. (2012). NF-κB, the first quarter-century: Remarkable progress and outstanding questions. Genes Dev..

[21] Ashburner B.P., Westerheide S.D., Baldwin A.S. (2001). The p65 (RelA) subunit of NF-κB interacts with the histone deacetylase (HDAC) corepressors HDAC1 and HDAC2 to negatively regulate gene expression. Mol. Cell Biol..

[22] Chen L.F., Greene W.C. (2004). Shaping the nuclear action of NF-κB. Nat. Rev. Mol. Cell Biol..

[23] Chen L.F., Williams S.A., Mu Y., Nakano H., Duerr J.M., Buckbinder L., Greene W.C. (2005). NF-κB RelA phosphorylation regulates RelA acetylation. Mol. Cell Biol..

[24] Campbell A.E., Ferraz Franco C., Su L.I., Corbin E.K., Perkins S., Kalyuzhnyy A., Jones A.R., Brownridge P.J., Perkins N.D., Eyers C.E. (2021). Temporal modulation of the NF-κB RelA network in response to different types of DNA damage. Biochem. J..

[25] Webster G.A., Perkins N.D. (1999). Transcriptional cross talk between NF-κB and p53. Mol. Cell Biol..

[26] Stark L.A., Dunlop M.G. (2005). Nucleolar sequestration of RelA (p65) regulates NF-κB-driven transcription and apoptosis. Mol. Cell Biol..

[27] Olson M.O., Dundr M., Szebeni A. (2000). The nucleolus: An old factory with unexpected capabilities. Trends Cell Biol..

[28] Grummt I. (2013). The nucleolus-guardian of cellular homeostasis and genome integrity. Chromosoma.

[29] Mayer C., Grummt I. (2005). Cellular stress and nucleolar function. Cell Cycle.

[30] van Sluis M., McStay B. (2017). Nucleolar reorganization in response to rDNA damage. Curr Opin Cell Biol..

[31] Boisvert F.M., van Koningsbruggen S., Navascues J., Lamond A.I. (2007). The multifunctional nucleolus. Nat. Rev. Mol. Cell Biol..

[32] Moore H.M., Bai B., Boisvert F.M., Latonen L., Rantanen V., Simpson J.C., Pepperkok R., Lamond A.I., Laiho M. (2011). Quantitative proteomics and dynamic imaging of the nucleolus reveal distinct responses to UV and ionizing radiation. Mol. Cell Proteomics.

[33] Andersen J.S., Lyon C.E., Fox A.H., Leung A.K., Lam Y.W., Steen H., Mann M., Lamond A.I. (2002). Directed proteomic analysis of the human nucleolus. Curr. Biol..

[34] Visintin R., Hwang E.S., Amon A. (1999). Cfi1 prevents premature exit from mitosis by anchoring Cdc14 phosphatase in the nucleolus. Nature.

[35] Latonen L. (2019). Phase-to-Phase With Nucleoli-Stress Responses, Protein Aggregation and Novel Roles of RNA. Front. Cell Neurosci..

[36] Frottin F., Schueder F., Tiwary S., Gupta R., Korner R., Schlichthaerle T., Cox J., Jungmann R., Hartl F.U., Hipp M.S. (2019). The nucleolus functions as a phase-separated protein quality control compartment. Science.

[37] Wang M., Bokros M., Theodoridis P.R., Lee S. (2019). Nucleolar Sequestration: Remodeling Nucleoli Into Amyloid Bodies. Front. Genet..

[38] Brecht R.M., Liu C.C., Beilinson H.A., Khitun A., Slavoff S.A., Schatz D.G. (2020). Nucleolar localization of RAG1 modulates V(D)J recombination activity. Proc. Natl. Acad. Sci. USA.

[39] Tao W., Levine A.J. (1999). P19(ARF) stabilizes p53 by blocking nucleo-cytoplasmic shuttling of Mdm2. Proc. Natl. Acad. Sci. USA.

[40] Holmberg Olausson K., Nister M., Lindstrom M.S. (2012). p53 -Dependent and -Independent Nucleolar Stress Responses. Cells.

[41] Mekhail K., Gunaratnam L., Bonicalzi M.E., Lee S. (2004). HIF activation by pH-dependent nucleolar sequestration of VHL. Nat. Cell Biol..

[42] Stark L.A., Din F.V.N., Zwacka R.M., Dunlop M.G. (2001). Aspirin-induced activation of the NF-kB signalling pathway: A novel mechanism for aspirin-mediated apoptosis in colon cancer cells. FASEB J..

[43] Loveridge C.J., Macdonald A.D., Thoms H.C., Dunlop M.G., Stark L.A. (2008). The proapoptotic effects of sulindac, sulindac sulfone and indomethacin are mediated by nucleolar translocation of the RelA(p65) subunit of NF-κB. Oncogene.

[44] Parrondo R., de las Pozas A., Reiner T., Rai P., Perez-Stable C. (2010). NF-κB activation enhances cell death by antimitotic drugs in human prostate cancer cells. Mol. Cancer.

[45] Sniderhan L.F., Garcia-Bates T.M., Burgart M., Bernstein S.H., Phipps R.P., Maggirwar S.B. (2009). Neurotrophin signaling through tropomyosin receptor kinases contributes to survival and proliferation of non-Hodgkin lymphoma. Exp. Hematol..

[46] Lee D.H., Forscher C., Di Vizio D., Koeffler H.P. (2015). Induction of p53-independent apoptosis by ectopic expression of HOXA5 in human liposarcomas. Sci. Rep..

[47] Dadsetan S., Balzano T., Forteza J., Agusti A., Cabrera-Pastor A., Taoro-Gonzalez L., Hernandez-Rabaza V., Gomez-Gimenez B., ElMlili N., Llansola M. (2016). Infliximab reduces peripheral inflammation, neuroinflammation, and extracellular GABA in the cerebellum and improves learning and motor coordination in rats with hepatic encephalopathy. J. Neuroinflamm..

[48] Audas T.E., Jacob M.D., Lee S. (2012). Immobilization of proteins in the nucleolus by ribosomal intergenic spacer noncoding RNA. Mol. Cell.

[49] Audas T.E., Jacob M.D., Lee S. (2012). The nucleolar detention pathway: A cellular strategy for regulating molecular networks. Cell Cycle.

[50] Arabi A., Rustum C., Hallberg E., Wright A.P. (2003). Accumulation of c-Myc and proteasomes at the nucleoli of cells containing elevated c-Myc protein levels. J. Cell Sci..

[51] Mattsson K., Pokrovskaja K., Kiss C., Klein G., Szekely L. (2001). Proteins associated with the promyelocytic leukemia gene product (PML)-containing nuclear body move to the nucleolus upon inhibition of proteasome-dependent protein degradation. Proc. Natl. Acad. Sci. USA.

[52] Latonen L. (2011). Nucleolar aggresomes as counterparts of cytoplasmic aggresomes in proteotoxic stress. Proteasome inhibitors induce nuclear ribonucleoprotein inclusions that accumulate several key factors of neurodegenerative diseases and cancer. Bioessays.

[53] Liu Y., Wang Y., Yang L., Sun F., Li S., Wang Y., Zhang G.A., Dong T., Zhang L.L., Duan W. (2021). The nucleolus functions as the compartment for histone H2B protein degradation. iScience.

[54] Thoms H.C., Loveridge C.J., Simpson J., Clipson A., Reinhardt K., Dunlop M.G., Stark L.A. (2010). Nucleolar targeting of RelA(p65) is regulated by COMMD1-dependent ubiquitination. Cancer Res..

[55] Maine G.N., Mao X., Komarck C.M., Burstein E. (2007). COMMD1 promotes the ubiquitination of NF-κB subunits through a cullin-containing ubiquitin ligase. EMBO J..

[56] Burstein E., Hoberg J.E., Wilkinson A.S., Rumble J.M., Csomos R.A., Komarck C.M., Maine G.N., Wilkinson J.C., Mayo M.W., Duckett C.S. (2005). COMMD proteins, a novel family of structural and functional homologs of MURR1. J. Biol. Chem..

[57] Mao X., Gluck N., Li D., Maine G.N., Li H., Zaidi I.W., Repaka A., Mayo M.W., Burstein E. (2009). GCN5 is a required cofactor for a ubiquitin ligase that targets NF-κB/RelA. Genes Dev..

[58] Riera-Romo M. (2018). COMMD1: A Multifunctional Regulatory Protein. J. Cell Biochem..

[59] Bartuzi P., Hofker M.H., van de Sluis B. (2013). Tuning NF-κB activity: A touch of COMMD proteins. Biochim. Biophys. Acta.

[60] O’Hara A., Simpson J., Morin P., Loveridge C.J., Williams A.C., Novo S.M., Stark L.A. (2014). p300-mediated acetylation of COMMD1 regulates its stability, and the ubiquitylation and nucleolar translocation of the RelA NF-κB subunit. J. Cell Sci..

[61] Ehm P., Nalaskowski M.M., Wundenberg T., Jucker M. (2015). The tumor suppressor SHIP1 colocalizes in nucleolar cavities with p53 and components of PML nuclear bodies. Nucleus.

[62] Vilotti S., Codrich M., Dal F.M., Pinto M., Ferrer I., Collavin L., Gustincich S., Zucchelli S. (2012). Parkinson’s disease DJ-1 L166P alters rRNA biogenesis by exclusion of TTRAP from the nucleolus and sequestration into cytoplasmic aggregates via TRAF6. PLoS ONE.

[63] Latonen L., Moore H.M., Bai B., Jaamaa S., Laiho M. (2011). Proteasome inhibitors induce nucleolar aggregation of proteasome target proteins and polyadenylated RNA by altering ubiquitin availability. Oncogene.

[64] Souquere S., Weil D., Pierron G. (2015). Comparative ultrastructure of CRM1-Nucleolar bodies (CNoBs), Intranucleolar bodies (INBs) and hybrid PML/p62 bodies uncovers new facets of nuclear body dynamic and diversity. Nucleus.

[65] Elson E.L. (2011). Fluorescence correlation spectroscopy: Past, present, future. Biophys J..

[66] Sanchez-Martin P., Saito T., Komatsu M. (2019). p62/SQSTM1: ‘Jack of all trades’ in health and cancer. FEBS J..

[67] Lamark T., Svenning S., Johansen T. (2017). Regulation of selective autophagy: The p62/SQSTM1 paradigm. Essays Biochem..

[68] Bjorkoy G., Lamark T., Brech A., Outzen H., Perander M., Overvatn A., Stenmark H., Johansen T. (2005). p62/SQSTM1 forms protein aggregates degraded by autophagy and has a protective effect on huntingtin-induced cell death. J. Cell Biol..

[69] Pankiv S., Lamark T., Bruun J.A., Overvatn A., Bjorkoy G., Johansen T. (2010). Nucleocytoplasmic shuttling of p62/SQSTM1 and its role in recruitment of nuclear polyubiquitinated proteins to promyelocytic leukemia bodies. J. Biol. Chem..

[70] Salmina K., Huna A., Inashkina I., Belyayev A., Krigerts J., Pastova L., Vazquez-Martin A., Erenpreisa J. (2017). Nucleolar aggresomes mediate release of pericentric heterochromatin and nuclear destruction of genotoxically treated cancer cells. Nucleus.

[71] Feng Y., Duan T., Du Y., Jin S., Wang M., Cui J., Wang R.F. (2017). LRRC25 Functions as an Inhibitor of NF-κB Signaling Pathway by Promoting p65/RelA for Autophagic Degradation. Sci. Rep..

[72] Khandelwal N., Simpson J., Taylor G., Rafique S., Whitehouse A., Hiscox J., Stark L.A. (2011). Nucleolar NF-κB/RelA mediates apoptosis by causing cytoplasmic relocalization of nucleophosmin. Cell Death. Differ..

[73] Lindstrom M.S. (2011). NPM1/B23: A Multifunctional Chaperone in Ribosome Biogenesis and Chromatin Remodeling. Biochem. Res. Int..

[74] Lin J., Kato M., Nagata K., Okuwaki M. (2017). Efficient DNA binding of NF-κB requires the chaperone-like function of NPM1. Nucleic Acids Res..

[75] Rao C., Liu B., Huang D., Chen R., Huang K., Li F., Dong N. (2021). Nucleophosmin contributes to vascular inflammation and endothelial dysfunction in atherosclerosis progression. J. Thorac. Cardiovasc. Surg..

[76] Wang Z., Gall J.M., Bonegio R., Havasi A., Illanes K., Schwartz J.H., Borkan S.C. (2013). Nucleophosmin, a critical Bax cofactor in ischemia-induced cell death. Mol. Cell Biol..

[77] Thompson J., Finlayson K., Salvo-Chirnside E., MacDonald D., McCulloch J., Kerr L., Sharkey J. (2008). Characterisation of the Bax-nucleophosmin interaction: The importance of the Bax C-terminus. Apoptosis.

[78] Kerr L.E., Birse-Archbold J.L., Short D.M., McGregor A.L., Heron I., Macdonald D.C., Thompson J., Carlson G.J., Kelly J.S., McCulloch J. (2007). Nucleophosmin is a novel Bax chaperone that regulates apoptotic cell death. Oncogene.

[79] Hochrainer K., Racchumi G., Zhang S., Iadecola C., Anrather J. (2012). Monoubiquitination of nuclear RelA negatively regulates NF-κB activity independent of proteasomal degradation. Cell. Mol. Life Sci..

[80] Hernandez-Verdun D. (2006). Nucleolus: From structure to dynamics. Histochem. Cell Biol..

[81] Yuan X., Zhao J., Zentgraf H., Hoffmann-Rohrer U., Grummt I. (2002). Multiple interactions between RNA polymerase I, TIF-IA and TAF(I) subunits regulate preinitiation complex assembly at the ribosomal gene promoter. EMBO Rep..

[82] Grummt I. (2003). Life on a planet of its own: Regulation of RNA polymerase I transcription in the nucleolus. Genes Dev..

[83] Pfister A.S. (2019). Emerging Role of the Nucleolar Stress Response in Autophagy. Front. Cell NeuroSci..

[84] Lindstrom M.S., Jurada D., Bursac S., Orsolic I., Bartek J., Volarevic S. (2018). Nucleolus as an emerging hub in maintenance of genome stability and cancer pathogenesis. Oncogene.

[85] Hiscox J.A., Whitehouse A., Matthews D.A. (2010). Nucleolar proteomics and viral infection. Proteomics.

[86] Rubbi C.P., Milner J. (2003). Disruption of the nucleolus mediates stabilization of p53 in response to DNA damage and other stresses. EMBO J..

[87] James A., Wang Y., Raje H., Rosby R., DiMario P. (2014). Nucleolar stress with and without p53. Nucleus.

[88] Woods S.J., Hannan K.M., Pearson R.B., Hannan R.D. (2015). The nucleolus as a fundamental regulator of the p53 response and a new target for cancer therapy. Biochim. Biophys. Acta.

[89] Russo A., Russo G. (2017). Ribosomal Proteins Control or Bypass p53 during Nucleolar Stress. Int. J. Mol. Sci..

[90] Pecoraro A., Virgilio A., Esposito V., Galeone A., Russo G., Russo A. (2020). uL3 Mediated Nucleolar Stress Pathway as a New Mechanism of Action of Antiproliferative G-quadruplex TBA Derivatives in Colon Cancer Cells. Biomolecules.

[91] Russo A., Esposito D., Catillo M., Pietropaolo C., Crescenzi E., Russo G. (2013). Human rpL3 induces G(1)/S arrest or apoptosis by modulating p21 (waf1/cip1) levels in a p53-independent manner. Cell Cycle.

[92] Bodem J., Dobreva G., Hoffmann-Rohrer U., Iben S., Zentgraf H., Delius H., Vingron M., Grummt I. (2000). TIF-IA, the factor mediating growth-dependent control of ribosomal RNA synthesis, is the mammalian homolog of yeast Rrn3p. EMBO Rep..

[93] Jin R., Zhou W. (2016). TIF-IA: An oncogenic target of pre-ribosomal RNA synthesis. Biochim. Biophys. Acta.

[94] Nguyen le X.T., Mitchell B.S. (2013). Akt activation enhances ribosomal RNA synthesis through casein kinase II and TIF-IA. Proc. Natl. Acad. Sci. USA.

[95] Bierhoff H., Dundr M., Michels A.A., Grummt I. (2008). Phosphorylation by casein kinase 2 facilitates rRNA gene transcription by promoting dissociation of TIF-IA from elongating RNA polymerase I. Mol. Cell Biol..

[96] Szymanski J., Mayer C., Hoffmann-Rohrer U., Kalla C., Grummt I., Weiss M. (2009). Dynamic subcellular partitioning of the nucleolar transcription factor TIF-IA under ribotoxic stress. Biochim. Biophys. Acta.

[97] Yuan X., Zhou Y., Casanova E., Chai M., Kiss E., Grone H.J., Schutz G., Grummt I. (2005). Genetic inactivation of the transcription factor TIF-IA leads to nucleolar disruption, cell cycle arrest, and p53-mediated apoptosis. Mol. Cell.

[98] Kreiner G., Bierhoff H., Armentano M., Rodriguez-Parkitna J., Sowodniok K., Naranjo J.R., Bonfanti L., Liss B., Schutz G., Grummt I. (2013). A neuroprotective phase precedes striatal degeneration upon nucleolar stress. Cell Death Differ..

[99] Parlato R., Kreiner G., Erdmann G., Rieker C., Stotz S., Savenkova E., Berger S., Grummt I., Schutz G. (2008). Activation of an endogenous suicide response after perturbation of rRNA synthesis leads to neurodegeneration in mice. J. NeuroSci..

[100] Thoms H.C., Dunlop M.G., Stark L.A. (2007). p38-mediated inactivation of cyclin D1/cyclin-dependent kinase 4 stimulates nucleolar translocation of RelA and apoptosis in colorectal cancer cells. Cancer Res..

[101] Fatyol K., Grummt I. (2008). Proteasomal ATPases are associated with rDNA: The ubiquitin proteasome system plays a direct role in RNA polymerase I transcription. Biochim. Biophys. Acta.

[102] Bailly A., Perrin A., Bou Malhab L.J., Pion E., Larance M., Nagala M., Smith P., O’Donohue M.F., Gleizes P.E., Zomerdijk J. (2016). The NEDD8 inhibitor MLN4924 increases the size of the nucleolus and activates p53 through the ribosomal-Mdm2 pathway. Oncogene.

[103] Kato T., Delhase M., Hoffmann A., Karin M. (2003). CK2 Is a C-Terminal IκB Kinase Responsible for NF-κB Activation during the UV Response. Mol. Cell.

[104] Jiang H.Y., Wek R.C. (2005). GCN2 phosphorylation of eIF2alpha activates NF-κB in response to UV irradiation. Biochem. J..

[105] Jiang H.Y., Wek S.A., McGrath B.C., Scheuner D., Kaufman R.J., Cavener D.R., Wek R.C. (2003). Phosphorylation of the alpha subunit of eukaryotic initiation factor 2 is required for activation of NF-κB in response to diverse cellular stresses. Mol. Cell Biol..

[106] DuRose J.B., Scheuner D., Kaufman R.J., Rothblum L.I., Niwa M. (2009). Phosphorylation of eukaryotic translation initiation factor 2alpha coordinates rRNA transcription and translation inhibition during endoplasmic reticulum stress. Mol. Cell Biol..

[107] Russo A., Maiolino S., Pagliara V., Ungaro F., Tatangelo F., Leone A., Scalia G., Budillon A., Quaglia F., Russo G. (2016). Enhancement of 5-FU sensitivity by the proapoptotic rpL3 gene in p53 null colon cancer cells through combined polymer nanoparticles. Oncotarget.

[108] Russo A., Saide A., Cagliani R., Cantile M., Botti G., Russo G. (2016). rpL3 promotes the apoptosis of p53 mutated lung cancer cells by down-regulating CBS and NF-κB upon 5-FU treatment. Sci. Rep..

[109] Wan F., Anderson D.E., Barnitz R.A., Snow A., Bidere N., Zheng L., Hegde V., Lam L.T., Staudt L.M., Levens D. (2007). Ribosomal protein S3: A KH domain subunit in NF-κB complexes that mediates selective gene regulation. Cell.

[110] Beji S., D’Agostino M., Gambini E., Sileno S., Scopece A., Vinci M.C., Milano G., Melillo G., Napolitano M., Pompilio G. (2021). Doxorubicin induces an alarmin-like TLR4-dependent autocrine/paracrine action of Nucleophosmin in human cardiac mesenchymal progenitor cells. BMC Biol..

[111] Chandra T., Kirschner K. (2016). Chromosome organisation during ageing and senescence. Curr Opin Cell Biol..

[112] Acosta J.C., Banito A., Wuestefeld T., Georgilis A., Janich P., Morton J.P., Athineos D., Kang T.W., Lasitschka F., Andrulis M. (2013). A complex secretory program orchestrated by the inflammasome controls paracrine senescence. Nat. Cell Biol..

[113] Tiku V., Antebi A. (2018). Nucleolar Function in Lifespan Regulation. Trends Cell Biol..

[114] Buchwalter A., Hetzer M.W. (2017). Nucleolar expansion and elevated protein translation in premature aging. Nat. Commun.

[115] Rosete M., Padros M.R., Vindrola O. (2007). The nucleolus as a regulator of cellular senescence. Medicina (B Aires).

[116] Nishimura K., Kumazawa T., Kuroda T., Katagiri N., Tsuchiya M., Goto N., Furumai R., Murayama A., Yanagisawa J., Kimura K. (2015). Perturbation of ribosome biogenesis drives cells into senescence through 5S RNP-mediated p53 activation. Cell Rep..

[117] Osorio F.G., Soria-Valles C., Santiago-Fernandez O., Freije J.M., Lopez-Otin C. (2016). NF-κB signaling as a driver of ageing. Int. Rev. Cell Mol. Biol..

[118] Din F.V., Theodoratou E., Farrington S.M., Tenesa A., Barnetson R.A., Cetnarskyj R., Stark L., Porteous M.E., Campbell H., Dunlop M.G. (2010). Effect of aspirin and NSAIDs on risk and survival from colorectal cancer. Gut.

[119] Burn J., Sheth H., Elliott F., Reed L., Macrae F., Mecklin J.P., Moslein G., McRonald F.E., Bertario L., Evans D.G. (2020). Cancer prevention with aspirin in hereditary colorectal cancer (Lynch syndrome), 10-year follow-up and registry-based 20-year data in the CAPP2 study: A double-blind, randomised, placebo-controlled trial. Lancet.

[120] Walker J., Hutchison P., Ge J., Zhao D., Wang Y., Rothwell P.M., Gaziano J.M., Chan A., Burn J., Chia J. (2018). Aspirin: 120 years of innovation. A report from the 2017 Scientific Conference of the International Aspirin Foundation, 14 September 2017, Charite, Berlin. Ecancermedicalscience.

[121] Chen J., Stark L.A. (2017). Aspirin Prevention of Colorectal Cancer: Focus on NF-κB Signalling and the Nucleolus. Biomedicines.

[122] van De S.B., Mao X., Zhai Y., Groot A.J., Vermeulen J.F., van der W.E., van Diest P.J., Hofker M.H., Wijmenga C., Klomp L.W. (2010). COMMD1 disrupts HIF-1alpha/beta dimerization and inhibits human tumor cell invasion. J. Clin. Investig..

[123] Peltonen K., Colis L., Liu H., Trivedi R., Moubarek M.S., Moore H.M., Bai B., Rudek M.A., Bieberich C.J., Laiho M. (2014). A targeting modality for destruction of RNA polymerase I that possesses anticancer activity. Cancer Cell.

[124] Nunez Villacis L., Wong M.S., Ferguson L.L., Hein N., George A.J., Hannan K.M. (2018). New Roles for the Nucleolus in Health and Disease. Bioessays.

[125] Myant K.B., Cammareri P., McGhee E.J., Ridgway R.A., Huels D.J., Cordero J.B., Schwitalla S., Kalna G., Ogg E.L., Athineos D. (2013). ROS production and NF-κB activation triggered by RAC1 facilitate WNT-driven intestinal stem cell proliferation and colorectal cancer initiation. Cell Stem Cell.

